# Large Scale Survey Research with Older Adults/Persons with Disabilities in a Public Health Crisis

**DOI:** 10.1093/geroni/igab046.3621

**Published:** 2021-12-17

**Authors:** Sarah Dys, Hannah Huebner, Norma Carrillo- Van Tongeren, Courtney Sirk, Harold Urman, Cathy Coddington

**Affiliations:** 1 Vital Research, LLC, Los Angeles, California, United States; 2 Vital Research, LLC, Vital Research, California, United States

## Abstract

Best practice for measuring quality improvement and consumer satisfaction of health and human services for older adults and people with disabilities relies on in-person survey administration. This poster highlights adaptation strategies undertaken across three large-scale evaluation studies of program/service delivery conducted during the COVID-19 pandemic, necessitating a departure from in-person techniques: 1) Integrated Satisfaction Measurement for the Program of All-Inclusive Care for the Elderly (I-SAT-PACE), 2) National Core Indicators- Aging and Disabilities/Intellectual and Developmental Disabilities (NCI-AD/IDD), and 3) Assisted Living Resident Quality of Life (AL-QOL). Data collection for these projects occurred from September 2020 to August 2021, providing an opportunity to showcase project adaptation over the course of the pandemic. Using project implementation examples across 15 states and approximately 10,100 participants, we discuss implications for successful survey coordination, interviewer training, data collection, and participant/stakeholder engagement during a public health emergency. Strategies included pivoting to phone, Zoom, and paper-based data collection and increasing technical assistance for field staff and participants. Project teams were able to increase access to participation by implementing multimodal survey delivery, mitigate coronavirus exposure, continue collecting older adults and people with disabilities’ experiences, and compare results based on method of delivery. Technology barriers, field staff dropout, need for larger sample sizes, and inclusion of participants with dementia, hearing, and speech impairments present important tradeoffs to consider. These examples indicate it is possible to administer hybrid data collection methods across populations with varying cognitive and physical abilities without compromising data quality.

